# Prediction of cervical cancer recurrence using textural features extracted from ^18^F-FDG PET images acquired with different scanners

**DOI:** 10.18632/oncotarget.17856

**Published:** 2017-05-15

**Authors:** Sylvain Reuzé, Fanny Orlhac, Cyrus Chargari, Christophe Nioche, Elaine Limkin, François Riet, Alexandre Escande, Christine Haie-Meder, Laurent Dercle, Sébastien Gouy, Irène Buvat, Eric Deutsch, Charlotte Robert

**Affiliations:** ^1^ INSERM, U1030, F-94805, Villejuif, France; ^2^ Université Paris-Sud, Université Paris-Saclay, F-94270, Le Kremlin-Bicêtre, France; ^3^ Gustave Roussy, Université Paris-Saclay, Department of Radiotherapy, F-94805, Villejuif, France; ^4^ Gustave Roussy, Université Paris-Saclay, Department of Medical Physics, F-94805, Villejuif, France; ^5^ IMIV, CEA, INSERM, CNRS, Université Paris-Sud, Université Paris-Saclay, CEA-SHFJ, Orsay, France; ^6^ French Military Health Services Academy, Ecole du Val-de-Grâce, Paris, France; ^7^ Institut de Recherche Biomédicale des Armées, Bretigny-sur-Orge, France; ^8^ INSERM, U1015, F-94805, Villejuif, France; ^9^ Gustave Roussy, Université Paris-Saclay, Department of Nuclear Medicine and Endocrine Oncology, F-94805, Villejuif, France; ^10^ Gustave Roussy, Université Paris-Saclay, Department of Gynecologic Surgery, F-94805, Villejuif, France

**Keywords:** radiomics, cervical cancer, texture, PET imaging

## Abstract

**Objectives:**

To identify an imaging signature predicting local recurrence for locally advanced cervical cancer (LACC) treated by chemoradiation and brachytherapy from baseline ^18^F-FDG PET images, and to evaluate the possibility of gathering images from two different PET scanners in a radiomic study.

**Methods:**

118 patients were included retrospectively. Two groups (G1, G2) were defined according to the PET scanner used for image acquisition. Eleven radiomic features were extracted from delineated cervical tumors to evaluate: (i) the predictive value of features for local recurrence of LACC, (ii) their reproducibility as a function of the scanner within a hepatic reference volume, (iii) the impact of voxel size on feature values.

**Results:**

Eight features were statistically significant predictors of local recurrence in G1 (p < 0.05). The multivariate signature trained in G2 was validated in G1 (AUC=0.76, p<0.001) and identified local recurrence more accurately than SUV_max_ (p=0.022). Four features were significantly different between G1 and G2 in the liver. Spatial resampling was not sufficient to explain the stratification effect.

**Conclusion:**

This study showed that radiomic features could predict local recurrence of LACC better than SUV_max_. Further investigation is needed before applying a model designed using data from one PET scanner to another.

## INTRODUCTION

Cervical cancer is a significant cause of morbidity and mortality, being the fourth most common cancer in women worldwide and the sixth in Europe [1]. The five-year survival rate strongly depends on the stage, based on the International Federation of Gynecology and Obstetrics (FIGO) classification, at the time of diagnosis: from 93% for stage IA to 15% for stage IVb [2]. Castelnau-Marchand et al. studied clinical outcomes of chemoradiation followed by image guided adaptive brachytherapy for 225 patients with cervical cancers (65% with a FIGO stage ≥ IIB) and observed a local recurrence rate of 13% [3].

Several factors have been associated with the probability of local control, such as the tumor size at diagnosis, the volume of the high-risk clinical target volume at time of brachytherapy, or the overall treatment time. However, in the era of image guided adaptive brachytherapy, it remains necessary to refine the prediction of outcome, and more particularly to more thoroughly identify patients who are at high risk of local recurrence and who would require intensification of treatment, such as dose escalation or who would be candidates for clinical trials of radiosensitizing agents. On the opposite, it might be clinically relevant to identify patients with a lower risk of local relapse, and therefore who are less likely to benefit from dose escalation.

Medical imaging plays a key role in the initial evaluation and staging of patients, guiding subsequent treatment decisions. Magnetic Resonance Imaging (MRI) is the reference standard for the pre-therapeutic assessment of the T-stage of gynecological tumors due to the fact that the technique allows high resolution, high soft-tissue contrast and functional imaging [4]. ^18^F-FDG PET (18-Fluorodeoxyglucose Positron Emission Tomography) has proved to be more accurate for the detection of metastatic lymph nodes [5] and allows the evaluation of glucose consumption and metabolic activity within the tumor, which provides important prognostic information in patients treated with chemoradiation [6]. Literature showed accuracies of 76 to 90% for staging of Locally Advanced Cervical Cancer (LACC) using MRI [7], compared to 60 to 69% for CT (Computed Tomography) [8]. However, even if conventional semi-quantitative PET indices such as Standardized Uptake Value (SUV), Metabolic Volume (MV) or Total Lesion Glycolysis (TLG) have proved useful as prognostic factors in LACC [6, 9], more information extracted from images holds promise to further increase the prognostic value of PET.

“Radiomics” is a non-invasive method of quantitative analysis of high throughput imaging traits that was first introduced to decode genomic activity of tumors [10, 11] and then applied to all pathologies and imaging modalities [12, 13]. Radiomics has great potential to influence patient care [14], from aiding diagnosis and classification of tumors by stage or histology [15–17], through the prediction of responses to radiotherapy [18] or to chemotherapy [19]. This technique can also be used to guide radiation therapy [20, 21]. Among the features used in this approach, textural features are of particular interest to characterize tumor heterogeneity.

Several studies previously carried out PET texture analysis to predict recurrence and response to chemoradiation in LACC. In 2009, El Naqa et al. published a study in 14 patients [22], showing that textural features calculated from the co-occurrence matrix had a higher predictive power than SUV for determining chemo-radiation failure risk, and that a linear combination of two features led to an Area Under the ROC (Receiver Operating Characteristic) Curve (AUC) of 0.76 for the prediction of response to chemotherapy. The change of textural features during chemoradiation was investigated by Yang et al. [23], showing that several features calculated from the gray-level run length and zone size matrices were significantly different in the baseline and post-treatment images for complete metabolic responders (CMR), with p < 0.001. However, neither SUV indices nor textural features could differentiate CMR from partial metabolic responders or progressive tumors, possibly because of the low number of patients (n = 20). In a third study including 90 patients treated with chemoradiation, the same group demonstrated the better performance of four textural features to distinguish CMR vs. non-CMR (p < 0.05) compared to the performance of SUV [24].

The aim of our study was to identify a radiomic signature measured on baseline ^18^F-FDG PET images predictive of local recurrence after chemo-radiation and brachytherapy in LACC. A second objective was to assess the robustness of selected non-redundant textural features with respect to the PET scanner used in acquiring the images.

## RESULTS

### Textural features for predicting local recurrence

Mean tumor volumes were not significantly different between groups (p = 0.2), with 39.9 ± 26.1 mL for G1 and 39.0 ± 38.9 mL for G2. No significant differences were observed in the distribution of histology and stage between the groups (Table 1). A total of 39 cases of local recurrence (G1: 28, G2: 11) were clinically identified during the median follow-up time of 3 years.

**Table 1:** Patient characteristics

Characteristic	Value	Percent
*Age*	48.5 ± 11.1 years (range: 27.1-83.0 years)	
*FIGO stage*		
Ib1	6	5.1%
Ib2	30	25.4%
IIa	7	5.9%
IIb	61	51.7%
IIIa	0	0%
IIIb	9	7.6%
IVa	4	3.5%
IVb	1	0.8%
*Histology*		
Squamous carcinoma	96	81.4%
Adenocarcinoma	22	18.6%
*PET device*		
Siemens Biograph	79	66.9%
GE Discovery 690	39	33.1%

Eight features identified patients who later showed local tumor recurrence in G1 (Table 2): SUV_mean_, SUV_max_, SUV_peak_, MV, LGZE and HGZE (p < 0.05) and TLG, Entropy (p < 0.01). None of the computed features were significantly different between relapsing patients and non-relapsing patients in G2, but Entropy had the lowest p-value (p = 0.052) and the highest AUC (0.70). No feature could distinguish relapsing from non-relapsing patients in an artificially reduced cohort from G1 (to include the same number of patients as G2).

**Table 2:** P-values, AUC and C.I. between relapsing and non-relapsing patients in VOI-T

Index	VOI-T G1 (N = 79)			VOI-T G1 (N = 39)	VOI-T G2 (N = 39)		
	*p-value*	*AUC*	*95% C.I*.	*p-value*	*p-value*	*AUC*	*95% C.I*.
**SUV_mean_**	**0.025**	0.65	0.52-0.78	0.226	0.104	0.67	0.49-0.84
**SUV_max_**	**0.022**	0.66	0.53-0.79	0.260	0.104	0.67	0.49-0.84
**SUV_peak_**	**0.014**	0.67	0.54-0.80	0.226	0.199	0.63	0.45-0.81
**MV**	**0.021**	0.66	0.54-0.78	0.206	0.391	0.59	0.40-0.77
**TLG**	**0.001**	0.72	0.61-0.84	0.091	0.221	0.63	0.44-0.81
**Homogeneity**	0.202	0.59	0.45-0.72	0.425	0.142	0.65	0.48-0.83
**Entropy**	**0.004**	0.70	0.57-0.82	0.160	0.052	0.70	0.52-0.87
**SRE**	0.375	0.56	0.43-0.69	0.470	0.168	0.64	0.47-0.82
**LRE**	0.404	0.56	0.43-0.69	0.473	0.178	0.64	0.46-0.81
**LGZE**	**0.026**	0.65	0.52-0.78	0.262	0.091	0.67	0.50-0.84
**HGZE**	**0.026**	0.65	0.52-0.78	0.263	0.118	0.66	0.49-0.83

Bold p-values are significant

Using a stepwise model selection by AIC on all features, a 4-feature signature maximizing complementary information was identified in both groups (Equations 1 and 2).

$$\text{G1}:-11.27+3.57\cdot \text{Entropy}-1.02\cdot {\text{SUV}}_{\text{mean}}+0.72\cdot {\text{SUV}}_{\text{max}}-22.08\cdot \text{SRE}$$ (Eq.1)

$$\text{G2}:-13.83+1.19\cdot {\text{SUV}}_{\text{peak}}+12.70\cdot \text{Homeogeneity+}87.25\cdot \text{LGZE-}0.01.\text{HGZE}$$ (Eq.2)

**Figure 1:**
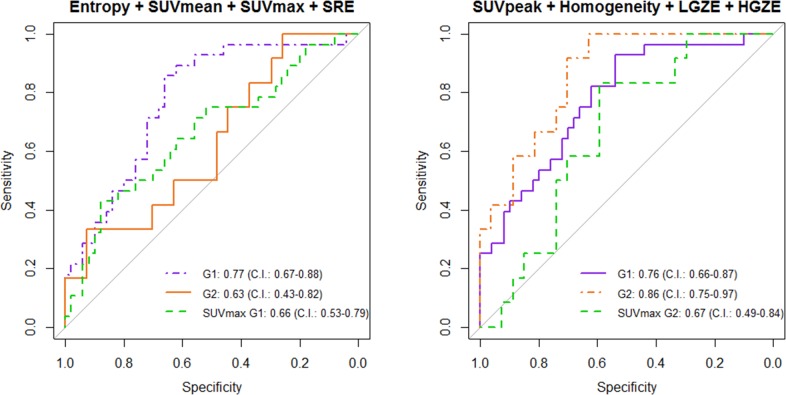
Multivariate analysis using G1 for training, G2 for validation (left) and G2 for training, G1 for validation (right) ROC curves of SUVmax are also presented.

The signature trained in G2 could differentiate non-relapsing and relapsing patients with AUC = 0.86 (C.I.: 0.75-0.97) in G2, and was validated in G1 with AUC = 0.76 (C.I.: 0.66-0.87). The performance of the signature trained in G1 was lower, with AUC = 0.77 (C.I.: 0.67-0.88) in G1 and AUC = 0.63 (C.I.: 0.43-0.82) in G2 (Figure 1). P-value from the Delong's test was statistically significant between the AUC of SUV_max_ and the AUC of the signature trained on G2, applied on both groups (p = 0.022 for G1, p = 0.030 for G2).

### Influence of PET characteristics and voxel size on texture feature values

**Figure 2:**
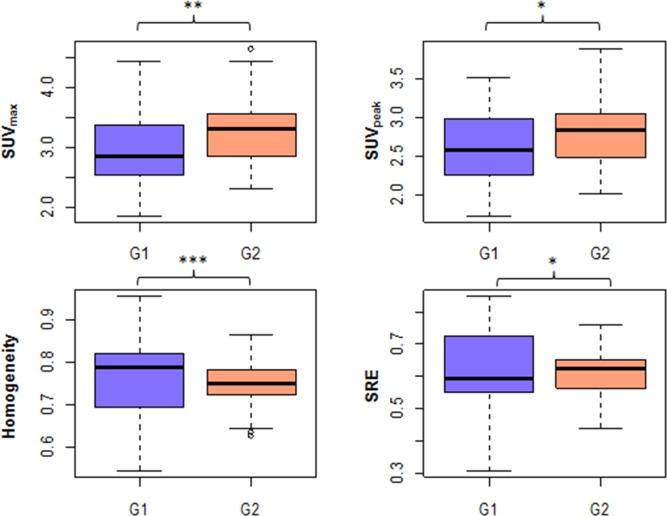
G1 vs. G2 in VOI-L for the 4 features that were significanly different between groups (original images) (*: 0.01<p<0.05, **: 0.001<p<0.01, ***: p<0.001)

Three patients were excluded from the whole cohort for liver analysis because no whole-body PET-CT image showing the liver was available. From original images, two conventional features (SUV_max_, SUV_peak_) and two textural features (Homogeneity, Entropy) calculated in the liver were significantly different between the two PET scanners according to Wilcoxon's test (Table 3, Figure 2).

**Table 3:** P-values between G1 and G2 in VOI-L, with original images, images resampled on G1 grid, on G2 grid, and on a 2 mm × 2 mm × 2 mm grid

Index	Original data	G1 grid size	G2 grid size	Resampling 2 mm
**SUV_mean_**	0.239	**0.038**	0.574	0.760
**SUV_max_**	**0.001**	**0.001**	**0.006**	**0.004**
**SUV_peak_**	**0.017**	**0.027**	0.138	0.087
**Homogeneity**	**0.031**	**<0.001**	**<0.001**	**<0.001**
**Entropy**	**<0.001**	**<0.001**	**<0.001**	**<0.001**
**SRE**	0.957	**<0.001**	**<0.001**	**<0.001**
**LRE**	0.967	**<0.001**	**<0.001**	**<0.001**
**LGZE**	0.504	0.118	0.638	0.742
**HGZE**	0.084	**0.021**	0.408	0.197

**Figure 3:**
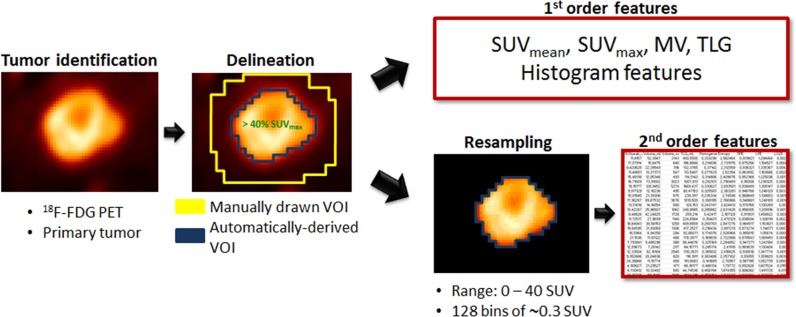
Radiomic feature extraction pipeline

The ability of image spatial resampling to remove the device-effect was also investigated on liver data. Neither resampling on G1 grid, G2 grid or on an isotropic grid of 2 mm side voxels could eliminate stratification effect. In all cases, at least five features were significantly different between the two cohorts (p < 0.001) (Table 3).

## DISCUSSION

### Prediction of recurrence

This study, performed in two cohorts of patients scanned with PET scanners having different properties, showed that several textural features were predictive of recurrence in a cohort scanned with the same PET machine (G1), and that a multivariate signature trained in G2 identified local recurrence with AUCs of 0.86 in the training set and 0.76 in the validation set. This signature was better than SUV_max_ when applied to both training and validation sets (p < 0.05). Although several studies discovered associations between tumor heterogeneity as reflected by PET texture indices and treatment outcomes, special attention should be paid to the methodology [25]. As shown in a study [26] comparing tumor delineation using the Nestle adaptive method [27] and a fixed threshold set to 40% of SUV_max_, different contours lead to differences in some textural feature values. As there is no consensus as to which segmentation method should be used, these authors studied the sensitivity of features to the segmentation and identified a list of robust features for three types of cancer. Further, the correlation between indices is of foremost importance. Starting from 41 indices calculated in three cohorts, they identified groups of highly correlated features (r > 0.80), some of which being highly correlated with MV [26]. In our study, only features selected for their robustness with respect to the segmentation and resampling methods were used for the processing.

Volumes of VOI-T were highly variable (39.8 ± 30.8 mL). As explained by Orlhac et al., the absolute resampling of SUV values avoids the volume dependence of textural features as opposed to relative resampling [28]. Another advantage of absolute resampling is the better distinction between tissues, and an intuitive variation in textural feature values [29]. Another team highlighted the impact of SUV discretization in such radiomic studies [30]. The authors showed that a fixed bin size (i.e. absolute resampling between two fixed boundaries) allows to obtain more comparable feature values between patients, although many teams used relative resampling with adaptive boundaries depending on minimum and maximum values in each tumor [23, 31]. In our study, eight features were identified as predictive of tumor local recurrence in G1 but not any in G2, possibly because of the low number of patients as suggested by results of univariate analysis in a subset of G1 including the same number of patients as in G2. Still, the highest AUC and the lowest p-value in G2 occurred for Entropy. AUC corresponding to Entropy were slightly higher than those found by Yang et al [24] in both groups (0.70 vs. 0.66), but the difference was not significant. In a previous study, Entropy calculated on PET images was significantly different between regions presenting different patterns of cells identified on pathological slices, suggesting that PET texture analysis captures the cellular heterogeneity of tumor and might provide additional information on tumor aggressiveness [32]. Unlike [24], our results showed a statistically significant correlation between SUV values and response to treatment, as it was previously reported [6, 33]. However, patient demographics were different between our study and [24], with their study having a higher number of advanced stages. Also, PET acquisition parameters and gray-level value resampling were different.

Regarding the identification of a signature for predicting recurrence, we chose to limit the number of parameters in the model by using AIC, a user-independent criterion for automatic model selection. A model containing too few parameters results in a low variance at the expense of high statistical bias for the fit parameters, whereas a model containing too many parameters may overfit the data, resulting in a low bias at the expense of high variance. We proved that a combination of a few radiomic features selected by an algorithm avoiding overlearning is predictive of treatment outcome. To our knowledge, this is the first study evaluating a combination of textural and SUV-based features computed on PET images to predict local recurrence of LACC with a validation set of images acquired on a separate device. Our results showed a difference between groups (Figure 1): the highest AUC was achieved using Entropy, SUV_mean_, SUV_max_ and SRE in G1 (AUC = 0.77), and using SUV_peak_, Homogeneity, LGZE and HGZE in G2 (AUC = 0.86). Highest AUCs were reached using G2 as training set and G1 as validation set (AUC = 0.86 in G2, AUC = 0.76 in G1), and the G2 signature predicted tumor recurrence significantly better than SUV_max_ in both sets. These results suggest that devices characterized by higher resolution and sensitivity are more sensitive to tissue heterogeneity and lead to more precise multivariate signature of recurrence. Other parameters such as injected activity, time per bed position, field of view could have likewise influenced the radiomic signature in favor of G2 PET device. An independent validation group will be necessary to strengthen the validity of this signature, but the modernization of PET scanners and the standardization of acquisition protocols through accreditations such as the EARL (European Association of nuclear medicine Research Ltd) FDG-PET/CT proposed by the European Association of Nuclear Medicine (EANM) are encouraging for performing multi-center PET radiomic studies such as suggested by Lasnon et al. [34].

### Device-dependence of radiomic features

In our study, we proposed a method to evaluate the variability of radiomic features between PET scanners, using patient data only. Both Entropy and SUV_max_ showed high differences between G1 and G2 in VOI-L with p ≤ 0.001. A recent article focused on the robustness of ^68^Ga-DOTANOC PET-based textural features when using various reconstruction settings on a single PET device to simulate a multicenter study [35]. Although the use of one PET device only may not reflect the full heterogeneity that may exist between multicenter scanners, the authors identified six parameters presenting less variability than SUV_max_ as a function of the reconstruction settings: Homogeneity, Entropy, Dissimilarity, HGRE (High Gray-level Run Emphasis), HGZE and ZP (Zone Percentage). Among these features, Homogeneity was highly robust with respect to the number of iterations, post-filtering level and reconstruction algorithm compared to SUV.

The impact of reconstruction settings was also investigated in retrospective studies [36, 37]. Yan et al. focused on the impact of Point Spread Function (PSF) modeling within the reconstruction, use of Time Of Flight (TOF) information, iteration number, grid size and Full Width at Half Maximum (FWHM) of the Gaussian filter on textural and first-order features [36]. Most features had a coefficient of variation (COV) lower than 20% across different reconstruction algorithms. A high COV was found for Homogeneity and SRE when varying grid size. This result is consistent with our study in VOI-L showing a high variability across the two devices. According to Yan et al., the grid size had the largest impact on feature values, whereas the FWHM and the iteration number had a lower impact. In our study, the analysis of VOI-L after resampling on G1 or G2 grid showed that stratification effect was not only due to voxel size. Same results were found using an isotropic grid of 2 mm × 2 mm × 2 mm voxel size. The technological differences between devices in terms of spatial resolution and sensitivity appear to be a limiting factor for applying a model derived using data from a PET scanner to data measured on a different PET scanner, or for pooling data from different scanners for model identification.

The device-dependence of radiomic features and their variability according to injected activity and acquisition parameters may explain the decrease in AUC between the training and validation sets. In the AIC stepwise algorithm, all 11 features were initially used to elaborate the multivariate signature, including those identified to be device-dependent according to the liver study (G1: SUV_max_, Entropy; G2: SUV_peak_, Homogeneity). In the literature, unlike CT studies including multicenter data [38], validation of PET radiomic signatures were mostly performed on subsets of patients from the initial cohort, acquired on the same device with the same acquisition parameters.

Therefore, it is necessary to investigate the reproducibility of radiomic features between devices. This comparison can be performed in a uniform ^18^F-FDG-filled phantom or in the healthy liver of patients. This latter method, used in our study, is particularly useful for retrospective studies where phantom images are no longer available.

There are several limitations in this study, especially its retrospective design and single center nature, which are sources of biases. Moreover, a higher local recurrence rate was observed in this cohort compared to previous cohorts from our institution [3]. This difference might be a consequence of scheduling considerations in local PET acquisitions, as the priority for nuclear imaging in our institution depends on the evolution and aggressiveness of the disease.

## MATERIALS AND METHODS

### Patient cohort

118 patients treated between 2005 and 2014 in our institution were retrospectively included in this study (Table 1). This project was reviewed and approved by the Institutional Review Board.

The inclusion criteria were as follows: (i) histologically-confirmed LACC, (ii) no surgery performed except for para-aortic lymph node dissection as surgical staging, (iii) no cervical conization performed before baseline PET-CT acquisition (due to the risk of inflammation), (iv) squamous carcinoma or adenocarcinoma histological subtypes, (v) minimum follow-up period of 15 months after external beam radiation therapy in patients without recurrence.

Treatment consisted of concurrent chemo-radiation followed by brachytherapy. 3D-conformationnal external beam radiotherapy was delivered in 25 daily fractions of 1.8 Gy each to reach a total dose of 45 Gy to the pelvis +/− the para-aortic area depending on the results of the primary para-aortic surgical staging. This was followed for all patients by a pulse-dose rate image-guided adaptive uterovaginal brachytherapy boost, delivering 15 Gy to 95% of the intermediate risk clinical target volume [3, 39]. Concomitant chemotherapy was systematically administered and the standard regimen was cisplatin 40 mg/m^2^ weekly, five times during external radiotherapy delivery, with a sixth cycle administered during brachytherapy. After treatment, patients were evaluated at 6 weeks using a pelvic MRI and a clinical examination. In case of complete response, they were then followed every 3 months during 3 years, then every 6 months during the following 2 years, and yearly thereafter. Biopsies were performed in case of non-metastatic MRI-based relapse suspicion.

### PET-CT acquisitions

Patients were divided in two groups, depending on the PET-CT device used for the baseline scan: a Siemens Biograph (Siemens AG, Erlangen, Germany) with LSO-based detectors (N = 79, G1) and a General Electric Discovery-690 (GE Healthcare, Waukesha, WI) with LYSO-based detectors (N = 39, G2). ^18^F-FDG injected activities were 5.9 ± 0.9 MBq/kg for G1 and 3.5 ± 0.1 MBq/kg for G2. Mean post-injection uptake time was 61.9 ± 6.4 min for G1 and 60.5 ± 5.8 min for G2. In G1, images were reconstructed using a 2D Ordered Subset Expectation Maximization (OSEM) algorithm (8 subsets, 2 iterations, no post-filtering). In G2, a fully 3D time-of-flight iterative reconstruction scheme (VUE Point FX) was used (OSEM algorithm, 24 subsets, 2 iterations) [40]. A low-dose CT scan was acquired in both sets for attenuation correction.

No PSF modeling was introduced in the reconstructions for both scanners. The voxel size was 5.3 mm × 5.3 mm × 3.4 mm (matrix size: 128 × 128, 4 min/bed position) for G1 and 2.7 mm × 2.7 mm × 3.4 mm (matrix size: 256 × 256, 2 min/bed position) for G2. PET images were converted in SUV units by normalization using the patient body weight.

### Radiomic pipeline

The entire radiomic feature extraction was performed using the LIFEx software (Local Image Feature Extraction, www.lifexsoft.org) [41]. The main steps of the radiomic pipeline are summarized in Figure 3.

The primary tumor was delineated on the PET images by a single observer (physicist, 3 years of experience) using a 40%-threshold of SUV_max_ (maximum SUV in the lesion) within a manually drawn volume (LIFEx software). The resulting volume from this semi-automatic segmentation was thereafter termed VOI-T (volume of interest-tumor). Special attention was paid to tumors located near the bladder wall due to the intense urinary uptake. VOI-T was systematically reviewed by a nuclear medicine physician (5 years of experience) and sometimes manually adjusted to exclude any biases due to bladder proximity and resulting partial volume effect.

For each VOI-T, five 1^st^-order features were extracted: SUV_mean_ (mean SUV in the VOI), SUV_max_, SUV_peak_ (mean SUV in a 1 mL sphere within the VOI such that the mean SUV in that sphere was maximum), MV, and TLG (product of SUV_mean_ and MV).

SUV values in VOI-T were then resampled in 128 discrete values using an absolute method in order to avoid the correlation between textural features and MV, reduce the impact of noise and the size of matrices. The minimum and maximum bounds of the resampling interval were set to 0 and 40 SUV leading to a bin size of 0.3 SUV (Equation 3). The higher bound was chosen to include all tumor SUV values [28].

$$\text{R}(\text{x})=\text{round }128\times \frac{1(x)}{40}$$ (Eq.3)

Three gray-level matrices were calculated in each VOI-T: the Gray-Level Co-occurrence Matrix (GLCM), the Gray-Level Run Length Matrix (GLRLM) and the Gray-Level Zone Length Matrix (GLZLM). Two methods were described in the literature to compute gray-level matrices in 3-dimensions [42]. In this study, GLCM and GLRLM were computed in 13 directions first to consider all independent directions between one voxel and its 26 neighbors [30]. Each textural feature extracted from these matrices corresponds finally to the average value over the 13 directions. Six textural indices (Homogeneity, Entropy from GLCM; Short-Run Emphasis (SRE), Long-Run Emphasis (LRE) from GLRLM; Low Gray-level Zone Emphasis (LGZE), High Gray-level Zone Emphasis (HGZE) from GLZLM) were analyzed as proposed by Orlhac et al. [26].

### Statistical analysis

All statistical analyses were performed using R software version 3.3.2.

First, a univariate analysis was performed to assess the ability of each individual feature for predicting local recurrence in each group separately. P-values of Wilcoxon's tests were computed between non-relapsing and relapsing patient features calculated in VOI-T for G1 and G2 separately. ROC analyses including AUC calculations were also performed to evaluate the performance of each feature using pROC library. 95% Confidence Intervals (C.I.) were computed using 2000 stratified bootstrap replicates [43]. To evaluate the influence of the patient number in univariate analysis, 100 random subsets of 39 patients from G1 were drawn so that the number of patients was identical to that in G2, and the mean p-value of each index was computed from all drawings.

Second, a multivariate analysis was performed using the original datasets to evaluate the added value of a combination of features for predicting local recurrence, and to develop a signature applicable in both groups. A stepwise model selection using the Akaike Information Criterion (AIC, library MASS) was applied to determine the best 4-feature multivariate signature for both groups [44, 45], successively used for training and validation of the model: first, G1 as training set and G2 as validation set, and secondly G2 as training set and G1 as validation set. The AIC is a measure of the relative quality of statistical models based on information theory. It allows comparison of the least-square fits of a given dataset obtained using several models of varying complexity. For a model *m*, with *N* the sample size, *K_m_* the number of parameters and *SS_m_* the sum of squares of the distances of the points from the model curve, the AIC of the model (*AIC_m_*) is defined as follows (Equation 4):

$${\text{AIC}}_{\text{m}}=\text{N In }\frac{{\text{SS}}_{\text{m}}}{\text{N}}+2({\text{K}}_{\text{m}}+1)$$ (Eq.4)

The model with the smallest AIC value is a compromise between goodness of fit on a given dataset and number of parameters.

Delong's test was performed between AUC of the 4-feature signature and AUC of SUV_max_ only for both groups.

### Influence of PET characteristics and voxel size on texture feature values

To evaluate the influence of the PET scanner on texture index values, a spherical volume of 75.5 mL for G1 and 75.7 mL for G2 was drawn in the liver (VOI-L). This region was supposed to be a homogeneous region of reference after systematic verification of the normal liver function [28]. Another parameter influencing textural feature values is the voxel size [29, 36]. G1 images were resampled on G2 grid (2.7 mm × 2.7 mm × 3.4 mm) and G2 images on G1 grid (5.3 mm × 5.3 mm × 3.4 mm) using bicubic interpolation. G1 and G2 images were also resampled to a common grid with a voxel size of 2 mm × 2 mm × 2 mm. The same radiomic pipeline as for VOI-T (Figure 3) was applied to VOI-L. Wilcoxon's tests were performed between G1 and G2 in VOI-L on native and on the three sets of resampled images to determine the extent to which technological differences can influence radiomic feature values and if spatial resampling is sufficient to remove the device dependence.

## CONCLUSIONS

In this study, we defined a 4-feature signature predicting local recurrence in LACC in two cohorts, and we validated the signature derived from the images acquired on the most recent scanner (G2) on the G1 group with AUC > 0.75 using radiomic features only. For both PET scanners, we showed that this signature predicted tumor recurrence better than SUV_max_.

We also demonstrated that it is challenging to merge images from two different PET scanners with different acquisition parameters without introducing bias due to differences between acquisition protocols. Multi-center or multi-device studies must thus be performed with caution, ensuring that biases are taken into account in the analyses.

## Abbreviations

AIC: Akaike information criterion
AUC: area under receiver operating characteristic (ROC) curve
CI: confidence interval
CMR: complete metabolic responders
COV: coefficient of variation
CT: computed tomography
EANM: European Association of Nuclear Medicine
EARL: European Association of nuclear medicine Research Ltd
18-FDG: 18-fluorodeoxyglucose
FIGO: International Federation of Gynecology and Obstetrics
FWHM: full width at half maximum
GLCM: Gray-Level Co-occurrence Matrix
GLRLM: Gray-Level Run Length Matrix
GLZLM: Gray-Level Zone Length Matrix
Gy: gray (unit of ionizing radiation dose)
HGRE: high gray-level run emphasis
HGZE: high gray-level zone emphasis
LACC: locally advanced cervical cancer
LSO: cerium doped lutetium oxyorthosilicate
LGZE: low gray-level zone emphasis
LIFEx: local image feature extraction
LRE: long-run emphasis
LYSO: cerium doped lutetium yttrium oxyorthosilicate
MRI: magnetic resonance imaging
MV: metabolic volume
OSEM: ordered subset expectation maximization
PET: positron emission tomography
PSF: point spread function
PDR: pulse dose rate
ROC: receiver operating characteristic
SRE: short-run emphasis
SUV: standardized uptake value
TLG: total lesion glycolysis
TOF: time of flight
VOI-L: liver volume of interest
VOI-T: tumor volume of interest
ZP: zone percentage
